# Perceived barriers to guideline adherence: A survey among general practitioners

**DOI:** 10.1186/1471-2296-12-98

**Published:** 2011-09-22

**Authors:** Marjolein Lugtenberg, Jako S Burgers, Casper F Besters, Dolly Han, Gert P Westert

**Affiliations:** 1 Scientific Centre for Care and Welfare (Tranzo), Tilburg University, PO Box 90153, 5000 LE Tilburg, the Netherlands; 2 Scientific Institute for Quality of Healthcare (IQ healthcare), Radboud University Nijmegen Medical Centre, PO Box 9101, 6500 HB Nijmegen, the Netherlands

## Abstract

**Background:**

Despite considerable efforts to promote and support guideline use, adherence is often suboptimal. Barriers to adherence vary not only across guidelines but also across recommendations within guidelines. The aim of this study was to assess the perceived barriers to guideline adherence among GPs by focusing on key recommendations within guidelines.

**Methods:**

We conducted a cross-sectional electronic survey among 703 GPs in the Netherlands. Sixteen key recommendations were derived from four national guidelines. Six statements were included to address the attitudes towards guidelines in general. In addition, GPs were asked to rate their perceived adherence (one statement) and the perceived barriers (fourteen statements) for each of the key recommendations, based on an existing framework.

**Results:**

264 GPs (38%) completed the questionnaire. Although 35% of the GPs reported difficulties in changing routines and habits to follow guidelines, 89% believed that following guidelines leads to improved patient care. Perceived adherence varied between 52 and 95% across recommendations (mean: 77%). The most perceived barriers were related to external factors, in particular patient ability and behaviour (mean: 30%) and patient preferences (mean: 23%). Lack of applicability of recommendations in general (mean: 22%) and more specifically to individual patients (mean: 25%) were also frequently perceived as barriers. The scores on perceived barriers differed largely between recommendations [minimum range 14%; maximum range 67%].

**Conclusions:**

Dutch GPs have a positive attitude towards the NHG guidelines, report high adherence rates and low levels of perceived barriers. However, the perceived adherence and perceived barriers varied largely across recommendations. The most perceived barriers across recommendations are patient related, suggesting that current guidelines do not always adequately incorporate patient preferences, needs and abilities. It may be useful to provide tools such as decision aids, supporting the flexible use of guidelines to individual patients in practice.

## Background

Clinical practice guidelines aim to improve the quality of patient care by providing specific recommendations for daily practice. Despite the considerable efforts in developing and implementing evidence-based guidelines, only a modest impact has been found on clinical practice [1-5]. A comprehensive study in the US showed that only about half of the patients (55%) received recommended care as described in the guidelines [6]. Similarly, in the Netherlands, general practitioners (GPs) do not optimally adhere to guidelines with adherence levels varying largely between practices and providers [7].

Many factors may influence the implementation of a guideline in practice. Barriers to guideline adherence can be related to the individual patient, the individual health care provider, the group of providers, the organisational context, and the social and cultural context of the healthcare system [8-10]. An adequate analysis of the barriers that prevent healthcare providers from using guidelines in practice has demonstrated to be an important initial step in improving guideline adherence and, subsequently, quality of care [8,9].

As different aspects of a guideline may provoke varying barriers, focusing on specific recommendations within guidelines may be useful in identifying barriers [11]. Several qualitative studies have focused on barriers at the level of key recommendations [11-14]. A focus group study among Dutch GPs showed that lack of applicability, organisational constraints, and lack of knowledge were the most prominent barriers to adherence to guidelines and that each individual key recommendation had a unique pattern of barriers [11].

Most studies focusing on barriers to specific recommendations in guidelines utilised qualitative designs with small sample sizes; large quantitative studies are thus far lacking. Whereas qualitative studies can provide detailed insight in the range of barriers that apply across recommendations in guidelines, quantitative studies are needed to quantify the prevalence of the barriers in a larger sample across the target group. The aim of our study is to quantitatively assess the attitudes of Dutch GPs towards guidelines and to assess the perceived barriers in adhering to key recommendations in guidelines. In addition, we explored the perceived adherence to key recommendations and hypothesised a reverse relationship between perceived adherence and perceived barriers.

## Methods

### Setting

In the Dutch healthcare system, the GP has a central role as a gate keeper to specialist and hospital care. Every Dutch citizen is obliged to register with a GP. GPs are reimbursed for their services by the patient's health insurance, which is obligatory for Dutch citizens. This results in Dutch general practices being highly accessible to patients. More than 90% of all newly encountered health problems are being managed within general practice, contributing to efficient, low-cost healthcare services [15]. Almost all GPs are members of the Dutch College of General Practitioners (NHG) [16], a body responsible for national guideline development and dissemination among their members. Currently, more than ninety guidelines have been developed and are updated regularly, covering the vast majority of acute and chronic conditions seen in general practice.

### Study population

We conducted an electronic survey among all GPs in the South Western part of the Netherlands (N = 703), using the mailing list of Stichting KOEL [17], a regional organisation supporting continuing medical education (CME) and practice management. After developing and pilot-testing the questionnaire, the final revision was sent to the GPs by an email linking to the electronic version of the questionnaire. They were offered one CME accreditation point (1 hour) for completing the questionnaire. A reminder was sent after two weeks and a second reminder after four weeks.

### Questionnaire

Before developing the questionnaire, we conducted a qualitative focus group study to gain an understanding of all the potential barriers that GPs may perceive in adhering to guidelines [11,14]. Twelve national guidelines were included covering a variety of conditions and diseases [11]. The barriers identified in the focus group discussions were classified in accordance to the framework of Cabana et al (1999) [10] (Table 1).

**Table 1:** Possible barriers to adhering to guideline recommendations in practice based on Cabana [10] and results from our focus groups study [11]

Knowledge related barriers	
*Lack of awareness/familiarity:*	GPs may be unaware of the (exact) content of the guideline recommendation

**Attitude related barriers**	

*Lack of agreement:*	GPs may disagree with the guideline recommendation due to perceived lack or inadequate interpretation of evidence or due to a lack of applicability of recommendations in general and more specifically to individual patients
*Lack of self-efficacy:*	GPs may believe that they cannot perform the guideline recommendation because they lack appropriate training or experience
*Lack of outcome expectancy:*	GPs may believe that even if they can perform the recommendation it will not affect patient outcomes
*Inertia of previous practice/lack of motivation:*	GPs may not follow recommendations because of difficulties of changing habits or old routines or lack of motivation

**External barriers**	

*Patient factors:*	GPs may be unable to reconcile patient preferences and demands with guideline recommendations or believe that patients are unable to perform the necessary action
*Guideline factors:*	GPs may believe that the guideline recommendations itself are unclear or ambiguous, incomplete, or too complex
*Environmental factors:*	GPs may be unable to overcome barriers in their practice environments, such as lack of time/time pressure, lack of resources/materials, organisational constraints within the own practice (e.g. arrangements with practice assistants), in other organisations (e.g. out of hours services, pharmacies) or between organisations (e.g. cooperation and arrangements with medical specialists) and lack of reimbursement

Subsequently, we selected four guidelines (eye inflammation (red eye), cerebrovascular accident (CVA), urinary tract infections (UTI), thyroid disorders) covering a diverse set of recommendations (diagnostic, treatment, referral etc) and representing both acute and chronic conditions. In designing the questionnaire, we used data from our focus group study in combination with an existing validated questionnaire to identify barriers to physician adherence to guidelines [18,19]. This was necessary as the existing questionnaire focused on barriers to guideline adherence in general rather than on barriers to adhering to specific key recommendations in guidelines.

In each questionnaire one out of four combinations of guidelines was included: 1. red eye and CVA; 2. red eye and UTI; 3. thyroid disorders and CVA or; 4. thyroid disorders and UTI. We distributed different combinations of guidelines to the GPs to make sure that the background characteristics of the GPs were evenly distributed across the different guidelines. A total of sixteen key recommendations were derived from these four guidelines (varying between three to five per guideline) (Additional file 1). Three of these recommendations concerned diagnosis, nine were treatment recommendations, two concerned referral, and two focused on education or rehabilitation.

Each of the questionnaires consisted of two sections: a general and guideline specific part. The general section included questions about demographics and professional characteristics such as age, type of practice and number of hours worked weekly. In addition, six statements on attitudes towards NHG guidelines in general were included, based on the framework of Cabana [10]. A 5-point Likert scale was used to rate the extent of agreement with the statements (ranging from 1. 'Strongly disagree' to 5. 'Strongly agree').

The guideline specific section consisted of statements on barriers to guideline adherence for the key recommendations of the two specific guidelines. For each of the key recommendations fifteen statements about barriers to guideline adherence were included. One of these statements concerned knowledge of the recommendation, seven focused on barriers related to attitude, and seven on external barriers. In addition to the barrier statements, one statement concerned the extent that GPs adhere to the recommendation in practice ('I follow this recommendation in practice'). Each statement was rated on the same 5-point Likert scale. For the statements concerning external barriers the option 'not applicable' was added to the response scale.

### Analysis

Descriptive statistics were used to describe the demographic and professional characteristics of the GPs (mean, standard deviation, percentages). In our analysis of the responses on the statements on attitudes towards guidelines in general, we grouped the scores 4 and 5 (agree/strongly agree), indicating agreement; the scores 3, indicating a neutral attitude, and the scores 1 and 2 (strongly/somewhat disagree), indicating disagreement.

Perceived adherence rates for each of the key recommendations were determined by calculating the percentage of respondents that either agreed or strongly agreed (score 4 and 5) to follow the recommendations in practice. To determine perceived barriers, we first recoded the barrier statements that were positively formulated, so that a higher score indicated a higher level of perceived barriers. Subsequently, we calculated the percentage of GPs that either agreed or strongly agreed (score 4 and 5) that a barrier was applicable for each of the key recommendations. The mean percentage refers to the mean percentage of GPs that perceive the different factors as barriers across all key recommendations.

To determine the association between perceived adherence and perceived barriers, we first calculated the mean percentage of respondents that agreed that barriers were applicable for knowledge related barriers, attitude related barriers, external barriers, and all barriers. Next, we calculated the Pearson Correlation between perceived adherence and each of the main categories of barriers and the total of all barriers.

## Results

We received 264 completed questionnaires, resulting in a response rate of 38% (264/703). The questionnaires distributed to the GPs yielded the following response for the four guidelines: 122 on red eye; 129 on thyroid disorder; 120 on CVA and 120 on UTI.

### Characteristics of GP sample

Table 2 summarizes the demographic and professional characteristics of the responding GPs. The majority of respondents were male (63%), most were aged between 55 and 64 years (37%), worked as independent GPs (80%), and worked in solo practices (37%). Comparing to the total population of Dutch GPs [20], GPs in the age group of 55-64 years were somewhat overrepresented in our sample.

**Table 2:** Demographic and professional characteristics of the responding GPs (n = 264)

	N	%	Mean (SD)	Total population of Dutch GPs^&^(%)
**Sex**				
Male	165	62.5		61.9
Female	99	37.5		38.1
**Age**			50.4 (8.9)	-
< 35	13	4.9		7.3
35-44	49	18.5		28.7
45-54	75	28.3		36.4
55-64	97	36.6		27.2
> 65	2	0.8		0.5
**Type of practice^+^**				
Solo	97	36.6		41.8
Partnered	85	32.1		31.3
Group	79	29.8		26.9
**Type of physician^+^**				
Independent	212	80.0		88.7
GP working for other GP	30	11.3		11.3
Flexible	10	3.8		^-^
Other	13	4.9		-
**Years working as GP**				
< 3	9	3.4		-
3-7	33	12.5		-
7-10	21	7.9		-
> 10	198	74.7		-

* Percentages may not add up to 100 due to missing variables

^&^Where independent and GP working for other GP only included: (8789 GPs: independent: 7799; GP working for other GP: 990).

^+^More than one answer possible

### General attitude towards guidelines

**Figure 1:**
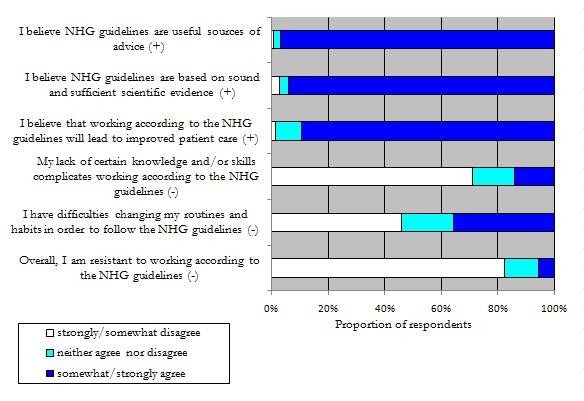
**GPs' ratings on statements measuring the attitude towards NHG guidelines in general (n = 260)**. White = Strongly/Somewhat disagree. Turquoise = Neither agree nor disagree. Blue = Somewhat/Strongly agree.

Almost all respondents (97%) agreed to the statement that NHG guidelines are useful sources of advice (Figure 1). In addition, 94% reported that they believed that NHG guidelines are based on sound and sufficient evidence.

Thirty-five percent of the GPs agreed to have difficulties changing their routines and habits in order to follow the NHG-guidelines. In addition 14% of the GPs indicated that their lack of knowledge or certain skills complicates working in accordance to the NHG guidelines.

### Perceived adherence

The mean perceived adherence rate across recommendations was 77% [SD: 15]. The guideline on red eye received the highest adherence rate (M = 83%; SD: 3.0); the guideline on CVA/stroke the lowest (M = 69%; SD: 15.2) (See Additional file 2 for scores on each of the key recommendations).

Recommendations on referral showed the highest rates of adherence (M = 94%; SD: 2.1), whereas recommendations on education or rehabilitation received the lowest rates of adherence (M = 57%; SD: 7.3). Recommendations on both diagnosing and treatment had intermediate rates of adherence (respectively 78%; SD: 12.8 and 77%; SD: 14.2).

Reported levels of adherence varied between 50 and 95% across the sixteen key recommendations. High levels of adherence were found for the key recommendations referral thyroid node (KR11; 95%) and treatment of thyroid hypo function (KR9; 94%). The recommendation on treatment of thyroid hyper function (KR10; 50%) and on education in CVA/stroke (KR8; 52%) received the lowest rates of perceived adherence.

### Perceived barriers and association with adherence

Table 3 summarises the percentage of respondents that agrees that specific barriers apply to specific recommendations. Overall, the mean percentage of GPs that agreed that barriers were applicable to the key recommendations varied from 4% (SD: 5.1) on lack of reimbursement to 30% (SD: 9.5) on patient ability and behaviour.

**Table 3:** Mean percentage of GPs that perceive various types of barriers, based on 16 recommendations from 4 guidelines (n = 264)

	Mean %	SD	Range	[min, max]
	***across all 16 recommendations***			
**Knowledge related barriers**				
Lack of awareness/familiarity	9.0	6.2	20.3	(1.7-22.0)
**Attitude related barriers**				
*Lack of agreement*				
Lack of evidence	12.2	3.8	13.6	(5.1-18.7)
Lack of applicability in general	22.4	13.4	42.5	(5.0-47.5)
Lack of applicability to patients	25.2	9.1	34.9	(11.0-45.9)
Lack of self-efficacy	10.8	13.0	49.6	(0.0-49.6)
Lack of outcome expectancy	9.6	5.3	17.7	(1.7-19.4)
Inertia of previous practice/lack of motivation	16.9	7.5	25.8	(3.9-29.7)
**External barriers**				
*Patient factors*				
Patient preferences	23.0	15.4	67.4	(8.8-76.2)
Patient ability and behaviour	29.7	9.5	33.6	(11.7-45.3)
*Guideline factors*				
Guideline recommendation factors	12.1	6.2	20.5	(2.4-22.9)
*Environmental factors*				
Lack of time/time pressure	12.7	14.1	51.7	(0.8-52.5)
Lack of resources/materials	6.1	7.4	23.3	(0.8-24.1)
Organisational constraints	13.9	9.1	30.1	(4.4-34.5)
Lack of reimbursement	3.8	5.1	15.2	(0.0-15.2)

Barriers related to knowledge received low scores with an average of 9% of the GPs perceiving lack of awareness/familiarity with the guideline recommendations as a barrier across the sixteen key recommendations (SD: 6.2). Among the barriers related to attitude, lack of applicability of the guideline in general (M = 22%; SD = 13.4) and more specifically to individual patients (M = 25%; SD: 9.1) had the highest score. The most perceived barriers were related to external factors, in particular patient ability and behaviour (M = 30%, SD: 9.5) and patient preferences (M = 23%; SD: 15.4). Lack of resources/materials and lack of reimbursement showed the lowest scores (M = 6%; SD: 7.4; and M = 4%; SD: 5.1 respectively).

The scores on perceived barriers differed largely between recommendations (See Additional file 2 for scores on each of the key recommendations and see Table 4 for examples of key recommendations and their specific barriers). The smallest range across recommendations was found for the barrier lack of evidence (14%) and the largest one for patient preferences (67%). Some barriers were widely applicable across recommendations (patient ability and behaviour, patient preferences, lack of applicability in general and to individual patients), whereas others received high scores for some recommendations only (lack of self-efficacy, inertia of previous practice/lack of motivation, guideline recommendation factors, lack of time/time pressure, lack of resources/materials, and organisational constraints). Other barriers received low scores across all recommendations (lack of awareness/familiarity with the recommendation, lack of outcome expectancy, lack of reimbursement).

**Table 4:** Examples of key recommendations and perceived barriers to adherence

Key recommendation 2 (Red eye): *Patients with a diffuse red eye, no itching, no alarming symptoms (pain, vision impairment, or photophobia), and no abnormalities of the cornea, have a likely diagnosis of infectious conjunctivitis. If the symptoms last shorter than 3 days or do not cause much discomfort, antibiotics are not necessary and a 'wait-and-see' strategy could be considered*.	

Most perceived barriers (> 35% of GPs) (n = 122):	Explanation:
- Patient preferences (76%)	GPs may believe that the guideline recommendation is difficult to reconcile with patient preferences and
- Lack of applicability to patients (46%)	demands, as patients often prefer, expect or demand antibiotics and do not rely on a 'wait-and-see' policy.
- Patient ability and behaviour (39%)	In relation to this, GPs may believe that the recommendation is difficult to apply in practice as it does
	not consider unique characteristics of patients or specific patient groups
**Key recommendation 8 (CVA):** *In the chronic phase of CVA (i.e. when no further improvements are to be expected) the GP provides information to the patients and their central caregivers with an emphasis on practical information that can contribute to a meaningful and satisfying daily life. They are also informed about activities of patient associations, peer groups, partner contacts, and educational meetings*.	

**Most perceived barriers (> 35% of GPs) (n = 120):**	**Explanation:**
- Lack of time/time pressure (53%)	GPs may believe that adhering to this recommendation is difficult due to additional work demands compared
- Lack of applicability general (48%)	to regular care. Therefore, they may think it is difficult to apply in practice. They may also believe that patients
- Patient ability and behaviour (45%)	are unable to comply with the necessary actions. Furthermore, organisational constraints such as lack of
- Organisational constraints (35%)	trained personnel or coordination with the activities performed by other healthcare providers
	(e.g. specialists in hospitals) make it difficult to apply the recommendation in practice.

**Key recommendation 10 (Thyroid disorder):** *If the GP has specific knowledge on thyroid disorders, patients with hyperthyroïd (Graves disease) could be treated using the 'combination method'. This includes full inhibition of the thyroid function with a thyreostatic (preferably thiamazole 1dd30 mg), and then providing levothyroxine. Discuss the pros and cons of the treatment options (medication, radioactive iodine, surgery) with the patient and involve him or her in decision making*.	

**Most perceived barriers (> 35% of GPs) (n = 129):**	**Explanation:**
- Lack of self-efficacy (50%)	GPs may not feel confident with performing the recommendation in practice, as they lack appropriate
- Lack of applicability (44%)	training or experience to treating patients with hyperthyroid. In relation to this, they may think that
	the recommendation is difficult to apply in practice

Adherence was negatively associated with the overall perceived barriers (-.82**). The strongest relation was found for attitude related barriers (-.86**), followed by external barriers (-.68**) and knowledge related barriers (-.67**).

## Discussion

This study illustrated that Dutch GPs have a positive attitude towards the national guidelines for general practice. In addition, they reported high rates of adherence and the perceived barriers were overall limited. However, rates of adherence and perceived barriers differed substantially across recommendations in guidelines. The most perceived barriers-that are widely applicable across recommendations-are patient related, suggesting that GPs believe that current guidelines do not always adequately incorporate patient preferences, needs and abilities.

GPs in our study had a positive attitude towards the NHG guidelines in general. Other studies focusing on physicians' attitudes towards guidelines [21,22] and in particular those of GPs [23,24], demonstrated overall positive attitudes as well. Moreover, the positive attitude found among our sample of Dutch GPs may be related to the fact that almost all GPs are a member of the NHG and that their guidelines are presented as 'guidelines for GPs developed by GPs'. This can result in a strong sense of ownership among the target group. Although the overall adherence rate reported by GPs was rather high, we further uncovered that the rates of adherence varied largely across recommendations. These findings are consistent with a comprehensive study based on data from medical records among 195 GPs working in 104 general practices in the Netherlands, showing that GPs overall adherence is about 74%, with levels of adherence varying largely between diagnoses [7].

In line with the overall positive attitude to guidelines and high rate of adherence, the reported barriers among our GPs were overall limited. Furthermore, we found a negative association between perceived adherence and all types of barriers; recommendations that were more adhered to in practice, received lower rates on barriers. We found that barriers related to knowledge were not perceived as a barrier, whereas some of the barriers related to attitude and external factors prevented GPs from applying recommendations consistently in practice. The most perceived barriers to adherence across key recommendations were patient ability and behaviour, patient preferences and lack of applicability in general and more specifically to individual patients. These findings suggest that GPs believe that preferences, abilities and needs of individual patients are not well incorporated in guidelines that focus on the 'average patient', complicating adherence to guideline recommendations in practice.

Other studies also indicated that lack of applicability can be a barrier to guideline adherence, particularly to patients with comorbidity [11,25]. That guidelines do in fact provide little guidance on the treatment of patients with comorbidities was confirmed in several studies [[26], Lugtenberg M, Burgers JS, Clancy C, Westert GP, Schneider EC: Current guidelines have limited applicability to patients with comorbid conditions: a systematic analysis of evidence-based guidelines, submitted]. Aside from comorbidities, generally, GPs can have difficulties balancing the needs of the individual patients with the aggregated needs of the population and deviate from guidelines by adjusting practice to the patients' individual needs [24]. To address these main barriers, it may not only be useful to involve patients in the process of guideline development [27-29], but also to adapt the guidelines to facilitate the integration of individual patients' preferences in clinical decision making [30]. It may be useful to provide tools such as decision aids to support the flexible use of guidelines to individual patients in practice.

Whereas lack of knowledge regarding guideline recommendations was mentioned as a barrier in the focus group study [11], it was not identified as a barrier in this study. Discrepancies between qualitative and quantitative studies have been found before and may be related to the superficial nature of a survey compared to the more problem-oriented focus in qualitative studies [24]. On the other hand, the aim of the focus group study was to identify the range of barriers, whereas the survey aimed to explore the relevance of the barriers among a larger sample of the target group. Other barriers that did not seem to be relevant across all recommendations were lack of evidence and lack of outcome expectancy, which is in line with the overall positive attitude of Dutch GPs towards NHG guidelines. Dutch GPs seem to value the NHG guidelines and do not question their scientific basis and content. Finally, lack of reimbursement was among the lowest scoring barriers. This may be related to the well-recognised role of GPs and appropriate financial structure within the Dutch healthcare system [31,32].

The main strength of our study is that we specifically focused on key recommendations in assessing adherence and barriers to guideline adherence. Our study shows that factors that influence adherence vary markedly across recommendations, resulting in specific patterns of barriers for individual key recommendations. It also shows that identifying barriers at the recommendation level is a useful approach. Some barriers may seem unimportant at guideline level, but appear to be very relevant for particular recommendations. A 'one size fits all approach' to guideline implementation will therefore be ineffective. Instead, interventions should be tailored to the barriers of specific recommendations. Although it is usually not feasible to develop interventions to address all barriers for all recommendations in guidelines, results from a detailed analysis may help in deciding where to focus the efforts. Also, substantial improvements can be achieved by focusing on barriers that are widely applicable across recommendations.

Some limitations of our study need to be mentioned. Although our response rate is only slightly below mean response rates of surveys among physicians [33,34], it may nevertheless limit the ability to generalise our findings. Those with a positive attitude towards guidelines may be overrepresented in our sample. To minimize this possible bias, we offered accreditation points for completing the questionnaire, creating an incentive to participate for all GPs. In addition, although GPs in the in the age group of 55-64 years were somewhat overrepresented, overall, our sample corresponded quite well with the total population of Dutch GPs in terms of basic characteristics [20]. Secondly, perceived barriers depend on GPs perceptions' of the situation and may not accurately reflect the (whole spectrum of) barriers. Similarly, perceived adherence rates may be subject to the phenomenon of social desirability, resulting in overestimations of adherence rates [35]. On the other hand, there are indications that self-reporting among physicians is a valid and reliable source for assessing clinical performance, with high levels of consistency with data from medical records [36].

Thirdly, we used an existing framework to classify the barriers. Whereas the use of a predefined framework is useful in analysing a wide range of barriers, the classification of barriers can also be disputed. Based on our qualitative focus group study [11] we suggest that lack of applicability should be a more prominent category, including different reasons such as patients with comorbidities and that patient factors should also include patients' abilities, needs and behaviour, rather than solely their preferences. Fourthly, our analysis of barriers is based on four guidelines, while GPs in the Netherlands currently have more than 90 guidelines at their disposal. The inclusion of other guidelines could potentially yielded different patterns of barriers. As a diverse set of recommendations of both acute and chronic conditions were included, we expect the identified barriers to be quite representative across all guidelines in general practice.

## Conclusions

Although Dutch GPs are generally positive about the NHG guidelines and report high adherence rates, rates of adherence and perceived barriers to adherence vary markedly across recommendations in guidelines. Guideline implementers should therefore focus on developing interventions that are tailored to the specific barriers of individual recommendations. Additionally, as patient related barriers were the most perceived barriers across recommendations, it may be useful to involve patients in the process of guideline development as well as in the actual decision making process. This could improve the applicability-and subsequently the implementability-of guideline recommendations in practice.

## Competing interests

The authors declare that they have no competing interests.

## Authors' contributions

ML participated in developing the questionnaire, analysing the data, and drafting and revising the manuscript. JB contributed in developing the questionnaire and critically revising the manuscript. CFB and DH made substantial contributions by analysing data and revising the manuscript. GW supervised the study, participated in the development of the questionnaire and critically revising the manuscript. All authors read and approved the final manuscript.

## Pre-publication history

The pre-publication history for this paper can be accessed here:

http://www.biomedcentral.com/1471-2296/12/98/prepub

## Supplementary Material

Additional file 1 **Key recommendations of guidelines (Dutch)**. Description of sixteen key-recommendations derived from four guidelines (in Dutch). Click here for file

Additional file 2 **Percentage of GPs that (strongly) agree to adhere to key recommendations in practice and that barriers are applicable to key recommendations from four guidelines**. Detailed description of scores on adherence and barriers for each of the sixteen key recommendations from four guidelines. Click here for file

## References

[B1] Grimshaw J Freemantle N Wallace S Russell I Hurwitz B Watt I Long A Sheldon T Developing and implementing clinical practice guidelines Qual Health Care 1995 4 55 64 10.1136/qshc.4.1.55 10142039 PMC1055269

[B2] Lugtenberg M Burgers JS Westert GP Effects of evidence-based clinical practice guidelines on quality of care: a systematic review Qual Saf Health Care 2009 18 385 392 10.1136/qshc.2008.028043 19812102

[B3] Grimshaw JM Russell IT Effect of clinical guidelines on medical practice: a systematic review of rigorous evaluations Lancet 1993 342 1317 1322 10.1016/0140-6736(93)92244-N 7901634

[B4] Grol R Improving the quality of medical care: building bridges among professional pride, payer profit, and patient satisfaction JAMA 2001 286 2578 2585 10.1001/jama.286.20.2578 11722272

[B5] Grimshaw J Eccles M Thomas R Maclennan G Ramsay C Fraser C Toward evidence-based quality improvement. Evidence (and its limitations) of the effectiveness of guideline dissemination and implementation strategies 1966-1998 J Gen Intern Med 2006 21 S14 S20 16637955 10.1111/j.1525-1497.2006.00357.x PMC2557130

[B6] McGlynn EA Asch SM Adams J Keesey J Hicks J DeCristofaro A Kerr EA The quality of health care delivered to adults in the united states N Engl J Med 2003 348 2635 2645 10.1056/NEJMsa022615 12826639

[B7] Braspenning J Schellevis F Grol R Tweede Nationale Studie naar ziekten en verrichtingen in de huisartspraktijk. Kwaliteit huisartsenzorg belicht. [The second Dutch National Survey of General Practice. Quality of primary healthcare discussed] 2004 Nijmegen/Utrecht: WOK/NIVEL

[B8] Grol R Beliefs and evidence in changing clinical practice BMJ 1997 315 418 421 9277610 10.1136/bmj.315.7105.418 PMC2127297

[B9] Grol R Grimshaw J From best evidence to best practice: effective implementation of change in patients' care Lancet 2003 362 1225 1230 10.1016/S0140-6736(03)14546-1 14568747

[B10] Cabana MD Rand CS Powe NR Wu AW Wilson MH Abboud PAC Rubin HR Why don't physicians follow clinical practice guidelines? A framework for improvement JAMA 1999 282 1458 1465 10.1001/jama.282.15.1458 10535437

[B11] Lugtenberg M Zegers-van Schaick JM Westert GP Burgers JS Why don't physicians adhere to guideline recommendations in practice? An analysis of barriers among Dutch general practitioners Implement Sci 2009 4 54 10.1186/1748-5908-4-54 19674440 PMC2734568

[B12] Schouten JA Hulscher MEJL Natsch S Kullberg B-J Van der Meer JWM Grol RPTM Barriers to optimal antibiotic use for community-acquired pneumonia at hospitals: a qualitative study Quality Saf Health Care 2007 16 143 149 10.1136/qshc.2005.017327 PMC2653154 17403764

[B13] Cabana MD Ebel BE Cooper-Patrick L Powe NR Rubin HR Rand CS Barriers pediatricians face when using asthma practice guidelines Arch Pediatr Adolesc Med 2000 154 685 693 10891020 10.1001/archpedi.154.7.685

[B14] Lugtenberg M Burgers JS Zegers-van Schaick JM Westert GP Guidelines on uncomplicated urinary tract infections are difficult to follow: perceived barriers and suggested interventions BMC Fam Pract 2010 11 51 10.1186/1471-2296-11-51 20584276 PMC2908068

[B15] Cardol M Van Dijk L De Jong JD De Bakker DH Westert GP Tweede Nationale Studie naar ziekten en verrichtingen in de huisartspraktijk: Huisartsenzorg: wat doet de poortwachter? [The second Dutch National Survey of General Practice: What does the gatekeeper do?] 2004 Utrecht, Bilthoven: NIVEL, Rijksinstituut voor Volksgezondheid en Milieu

[B16] Nederlands Huisartsen Genootschap (NHG) http://nhg.artsennet.nl

[B17] Stichting Kwaliteit en Opleiding Eerstelijnszorg (KOEL) http://www.stichtingkoel.nl

[B18] Peters MAJ Harmsen M Laurant MGH Wensing M Ruimte voor verandering? Knelpunten en mogelijkheden voor verbeteringen in de patiëntenzorg Room for change? Barriers and facilitators for improvements in patient care 2003 Nijmegen: Afdeling Kwaliteit van zorg (WOK), UMC St Radboud

[B19] Peters MAJ Harmsen M Laurant MGH Wensing M Barriers and facilitator assessment instrument: introduction, instructions and instrument 2005 Nijmegen: Afdeling Kwaliteit van zorg (WOK), UMC St Radboud

[B20] Hingstman L Kenens RJ Cijfers uit de registratie van huisartsen-peiling 2009. [Data from the registration of general practitioners 2009] 2009 Utrecht: NIVEL

[B21] Tunis SR Hayward RSA Wilson MC Rubin HR Bass EB Johnston M Steinberg EP Internists' Attitudes about Clinical Practice Guidelines Ann Intern Med 1994 120 956 8172440 10.7326/0003-4819-120-11-199406010-00008

[B22] Farquhar CM Kofa EW Slutsky JR Clinicians' attitudes to clinical practice guidelines: A systematic review MJA 2002 177 502 12405894 10.5694/j.1326-5377.2002.tb04920.x

[B23] Watkins C Harvey I Langley C Gray S Faulkner A General practitioners' use of guidelines in the consultation and their attitudes to them Br J Gen Pract 1999 49 11 14 10622009 PMC1313310

[B24] Carlsen B Bringedal B Attitudes to clinical guidelines--do GPs differ from other medical doctors? BMJ Qual Saf 2011 20 158 162 10.1136/bmjqs.2009.034249 21209148

[B25] Smith L Walker A Gilhooly K Clinical guidelines on depression: a qualitative study of GPs' views J Fam Pract 2004 53 556 561 15251095

[B26] Boyd CM Darer J Boult C Fried LP Boult L Wu AW Clinical practice guidelines and quality of care for older patients with multiple comorbid diseases: implications for pay for performance JAMA 2005 294 716 724 10.1001/jama.294.6.716 16091574

[B27] Crawford MJ Rutter D Manley C Weaver T Bhui K Fulop N Tyrer P Systematic review of involving patients in the planning and development of health care BMJ 2002 325 1263 10.1136/bmj.325.7375.1263 12458240 PMC136920

[B28] Schunemann H Fretheim A Oxman A Improving the use of research evidence in guideline development: 10. Integrating values and consumer involvement Health Res Policy Syst 2006 4 22 10.1186/1478-4505-4-22 17147811 PMC1697808

[B29] Krahn M Naglie G The Next Step in Guideline Development JAMA 2008 300 436 438 10.1001/jama.300.4.436 18647988

[B30] Van der Weijden T Legare F Boivin A Burgers J Van Veenendaal H Stiggelbout A Faber M Elwyn G How to integrate individual patient values and preferences in clinical practice guidelines? A research protocol Implement Sci 5 10 10.1186/1748-5908-5-10 PMC2824684 20205815

[B31] Faber M Burgers J Voerman G Grol R International Health Policy Survey 2010-Commonwealth Fund, Onderzoek onder burgers in 11 landen 2010 Nijmegen: IQ healthcare, UMC St Radboud

[B32] Faber MJ Burgers JS Grol R Westert GP Bell E WG, Merrick J Achieving high performance quality in primary healthcare: the Dutch example Translational research for primary healthcare New York: NOVA Publishers in press

[B33] Asch DA Jedrziewski MK Christakis NA Response rates to mail surveys published in medical journals J Clin Epidemiol 1997 50 1129 1136 10.1016/S0895-4356(97)00126-1 9368521

[B34] Grava-Gubins I Scott S Effects of various methodologic strategies: Survey response rates among Canadian physicians and physicians-in-training Can Fam Physician 2008 54 1424 1430 18854472 PMC2567275

[B35] Adams AS Soumerai SB Lomas J Ross-Degnan D Evidence of self-report bias in assessing adherence to guidelines Int J Qual Health Care 1999 11 187 192 10.1093/intqhc/11.3.187 10435838

[B36] Spies TH Mokkink HGA De Vries Robbé PF Grol RPTM Which data source in clinical performance assessment? A pilot study comparing self-recording with patient records and observation Int J Qual Health Care 2004 16 65 72 10.1093/intqhc/mzh001 15020562

