# Supplementary material for: Perceived barriers to guideline adherence: A survey among general practitioners
Source: BMC Fam Pract. 2011 Sep 22;12:98. doi: 10.1186/1471-2296-12-98 (PMC3197492; doi:10.1186/1471-2296-12-98)
Supplement: Additional file 1 — Key recommendations of guidelines (Dutch). Description of sixteen key-recommendations derived from four guidelines (in Dutch). [file 1471-2296-12-98-S1.DOC]

**Additional file 1: Key recommendations of guidelines (Dutch)**

**Guideline 1 Eye Inflammation (‘Red eye’)/ Het rode oog** (M57; 2006)

**Kernaanbeveling 1:**

Bij een rood oog gepaard gaande met pijn, daling van het gezichtsvermogen of lichtschuwheid (indien niet veroorzaakt door keratoconjunctivitis fotoelectrica, corpus alienum of ander trauma), wordt de visus bepaald, de pupillen en pupilreacties beoordeeld en nader onderzoek van de cornea verricht met behulp van fluoresceïnekleuring.

**Kernaanbeveling 2:**

Bij diffuse roodheid en afwezigheid van jeuk, alarmsymptomen (pijn, visusdaling of lichtschuwheid) en cornea-afwijkingen, is er waarschijnlijk sprake van een infectieuze conjunctivitis. Indien de klachten korter dan drie dagen duren of er bestaat niet veel hinder, kan worden afgewacht zonder antibiotische behandeling.

**Kernaanbeveling 3:**

Als bij een (vermoedelijk) bacteriële conjunctivitis (diffuse roodheid,’s ochtends dichtgeplakte ogen en afwezigheid van jeuk, alarmsymptomen en cornea-afwijkingen) wordt besloten tot antibiotische behandeling, gaat de voorkeur uit naar chlooramfenicol oogzalf 1% 2-4 dd. Bij een blefaritis kan ook fusidinezuur worden voorgeschreven, in andere gevallen van conjunctivitis is dit middel niet zinvol gezien de resistentieontwikkeling.

#### Guideline 2 Cerebrovascular accident / CVA (M81; 2004)

**Kernaanbeveling 4:**

Patiënten met een CVA (acute neurologische uitvalsverschijnselen, zich uitend in verlamming van ledematen of gelaat, spraakstoornissen, of anderszins) worden op korte termijn verwezen voor opname op een *stroke-unit*, tenzij de uitvalsverschijnselen slechts gering van omvang zijn of spontaan al sterk verbeteren.

**Kernaanbeveling 5:**

Bij patiënten met een CVA die thuisblijven omdat de uitvalsverschijnselen spontaan sterk verbeteren, wordt een cardiovasculair risicoprofiel opgesteld (zie NHG-Standaard Cardiovasculair risicomanagement). Tevens wordt direct gestart met acetylsalicylzuur 1dd 160 mg gedurende twee weken, waarna de dosering wordt verlaagd naar 1dd 80 mg.

**Kernaanbeveling 6**:

Bij patiënten met een CVA die thuisblijven omdat de uitvalsverschijnselen spontaan sterk verbeteren, wordt het voorschrijven van bloeddrukverlagende medicatie onmiddellijk na het CVA afgeraden. Het is beter daar twee weken mee te wachten tot de patiënt klinisch stabiel is.

**Kernaanbeveling 7:**

Bij patiënten met een CVA die thuisblijven, draagt de huisarts zorg voor een spoedige start van de revalidatie - in een verpleeghuis (dagbehandeling), revalidatiecentrum of thuis - en periodieke evaluatie van het beloop en de behoefte aan zorg.

#### Kernaanbeveling 8:

Ook in de chronische fase (d.w.z. als er geen verbetering meer te verwachten is) geeft de huisarts voorlichting aan patiënten met een CVA en hun centrale verzorgers met het accent op praktische informatie die kan bijdragen aan een zinvolle en bevredigende dagbesteding. Ook worden zij geattendeerd op activiteiten van de patiëntenvereniging zoals lotgenotencontacten, partnercontacten en voorlichtingsbijeenkomsten.

#### Guideline 3 Thyroid disorder/ Schildklieraandoeningen (M31; 2006)

**Kernaanbeveling 9:**

Streef bij de behandeling van een patiënt met **hypothyreoïdie** naar een normale TSH-waarde en stel de medicatie (met levothyroxine) verder bij op grond van klachten. Spreek in de instelfase, niet eerder dan zes weken na de laatste doseringsverandering, een laboratoriumcontrole (TSH en vrij T4) af. In het eerste jaar nadat de patiënt klachtenvrij is geworden en het TSH en vrije T4 door medicatie zijn genormaliseerd, vinden de controles elke drie maanden plaats. Spreek vervolgens een jaarlijkse controle af, levenslang.

**Kernaanbeveling 10:**

Indien de huisarts specifieke kennis heeft van schildklieraandoeningen kan een patiënt met **hyperthyreoïdie** (ziekte van Graves) door de huisarts medicamenteus worden behandeld via de combinatiemethode. Hierbij wordt de schildklier eerst volledig stilgelegd met een thyreostaticum (bij voorkeur thiamazol 1dd30 mg), waarna levothyroxine wordt bijgegeven. Bespreek de voor- en nadelen van de verschillende behandelopties (medicamenteus, radioactief jodium, chirurgie) met de patiënt en betrek hem of haar bij de besluitvorming.

**Kernaanbeveling 11**:

De huisarts verwijst patiënten met een solitaire nodus of met een dominante nodus in een multinodulair struma naar een internist voor aanvullende diagnos­tiek.

**Guideline 4 Urinary Tract Infections/ Urineweginfectie** (M05; 2005)

**Kernaanbeveling 12:**

Het urineonderzoek bij klinische verdenking op urineweginfecties bestaat in eerste instantie uit een nitriettest, waarna bij een negatieve uitslag een dipslide wordt ingezet.

**Kernaanbeveling 13:**

Ongecompliceerde urineweginfecties, d.w.z. urineweginfecties bij niet-zwangere, overigens gezonde vrouwen, dienen in eerste instantie behandeld te worden met nitrofurantoïne. Bij overgevoeligheid voor nitrofurantoïne wordt trimethoprim geadviseerd.

**Kernaanbeveling 14**:

Bij een gecompliceerde urineweginfectie, d.w.z bij tekenen van weefselinvasie (koorts, rillingen, algemeen ziek-zijn, flank- of perineumpijn) of urineweginfectie bij patiënten uit een risicogroep (mannen, zwangere vrouwen, personen jonger dan 12 jaar, patiënten met afwijkingen aan de nieren of urinewegen in de voorgeschiedenis (zoals ernstige nierinsufficiëntie, cystennieren, nierstenen, een neurogene blaas of bemoeilijkte mictie), patiënten met een verminderde weerstand (zoals tgv. bestraling, immunosuppressiva of diabetes mellitus), patiënten met een verblijfskatheter) dient voorafgaand aan de behandeling urine te worden verzameld voor kweek en resistentiebepaling.

**Kernaanbeveling 15**:

Bij een gecompliceerde urineweginfectie dienen patiënten **met** tekenen van weefselinvasie, evenals alle jongens tot 12 jaar, meisjes tot en met 4 jaar en daarnaast patiënten met aandoeningen van de nieren of urinewegen, een verminderde weerstand (m.u.v. diabeten) of een verblijfskatheter, gedurende 10 dagen te worden behandeld met amoxicilline/ clavulaanzuur, totdat de uitslag van de kweek en resistentiebepaling bekend is. Bij overgevoeligheid dient dit te worden vervangen door co-trimoxazol of een fluorchinolon (norfloxacine 2dd400 mg of ciprofloxacine 2dd500 mg, maar niet in de zwangerschap of tijdens lactatie of bij leeftijd < 16 jaar).

**Kernaanbeveling 16:**

Bij een gecompliceerde urineweginfectie bij patiënten zonder tekenen van weefselinvasie dienen mannen, zwangeren, meisjes van 5-12 jaar en diabeten gedurende 7 dagen te worden behandeld met nitrofurantoïne totdat de uitslag van de kweek en resistentiebepaling bekend is. Bij overgevoeligheid voor nitrofurantoïne wordt gekozen voor 7 dagen trimethoprim.
