# Supplementary material for: Perceived barriers to guideline adherence: A survey among general practitioners
Source: BMC Fam Pract. 2011 Sep 22;12:98. doi: 10.1186/1471-2296-12-98 (PMC3197492; doi:10.1186/1471-2296-12-98)
Supplement: Additional file 2 — Percentage of GPs that (strongly) agree to adhere to key recommendations in practice and that barriers are applicable to key recommendations from four guidelines. Detailed description of scores on adherence and barriers for each of the sixteen key recommendations from four guidelines. [file 1471-2296-12-98-S2.DOC]

**Additional file 2: Percentage of GPs that (strongly) agree to adhere to key recommendations in practice and that barriers are applicable to key recommendations from four guidelines**

|  | | **Adhe-rence** | |  | | **Knowledge related barriers** |  | **Attitude related barriers** | | | | | |  | **External barriers** | | | | | | |
| --- | --- | --- | --- | --- | --- | --- | --- | --- | --- | --- | --- | --- | --- | --- | --- | --- | --- | --- | --- | --- | --- |
|  | |  |  |  | |  |  | Lack of agreement | | |  |  |  |  | *Patient factors* | | *Guideline*  *factors* | *Environmental factors* | | | |
|  | |  |  |  | | Lack of awareness/ familiarity |  | Lack of evidence | Lack of applicability: general | Lack of applicability: to patient | Lack of self-efficacy | Lack of outcome expectancy | Inertia previous practice/ lack of motivation |  | Patient preferences | Patient ability/be-haviour | Guideline recommendation factors | Lack of time/ time pressure | Lack of resources/ materials | Organisa-  tional constraints | Lack of reimbursement |
| **Guideline Red eye (n=122)** | | **82.9** |  | | **7.0** | |  | **12.0** | **17.1** | **25.9** | **2.6** | **11.7** | **18.2** |  | **36.4** | **33.4** | **7.2** | **10.5** | **6.4** | **7.9** | **1.1** |
| KR 1: diagnosis | | 85.0 |  | | 11.0 | |  | 9.1 | 11.8 | 14.2 | 5.5 | 7.9 | 15.7 |  | 8.8 | 26.7 | 6.6 | 18.5 | 15.3 | 5.9 | 3.2 |
| KR 2: no antibiotics | | 79.5 |  | | 5.8 | |  | 13.5 | 31.2 | 45.9 | 2.4 | 13.9 | 19.7 |  | 76.2 | 39.3 | 8.5 | 12.3 | 3.2 | 13.4 | 0.0 |
| KR3: preferred antibiotic | | 84.2 |  | | 4.2 | |  | 13.3 | 8.3 | 17.5 | 0.0 | 13.4 | 19.2 |  | 24.1 | 34.2 | 6.4 | 0.8 | 0.8 | 4.4 | 0.0 |
| **Guideline CVA/Stroke (n=120)** | | **68.3** |  | | **14.7** | |  | **12.8** | **30.0** | **27.8** | **12.7** | **9.7** | **16.6** |  | **22.7** | **34.1** | **15.4** | **20.8** | **6.4** | **22.8** | **6.1** |
| KR4: refer to stroke unit | | 92.4 |  | | 3.3 | |  | 13.0 | 18.5 | 31.1 | 3.4 | 9.3 | 7.6 |  | 24.3 | 25.2 | 12.1 | 2.5 | 0.8 | 13.5 | 0.8 |
| KR5: risk profile and aspirin | | 64.1 |  | | 16.1 | |  | 18.7 | 14.6 | 33.3 | 6.9 | 17.1 | 16.2 |  | 23.0 | 30.7 | 14.1 | 9.4 | 0.9 | 16.3 | 0.9 |
| KR6: no blood pressure lowering drugs | | 71.2 |  | | 17.0 | |  | 9.7 | 12.7 | 19.5 | 5.9 | 7.6 | 10.1 |  | 20.3 | 24.5 | 7.6 | 4.2 | 2.5 | 15.3 | 3.4 |
| KR7: rehabilitation | | 61.9 |  | | 15.2 | |  | 11.9 | 41.5 | 28.8 | 13.5 | 7.6 | 19.5 |  | 28.8 | 44.9 | 20.3 | 35.6 | 7.6 | 34.5 | 10.2 |
| KR8: education | | 51.7 |  | | 22.0 | |  | 10.6 | 47.5 | 26.3 | 33.9 | 6.7 | 29.7 |  | 17.0 | 45.3 | 22.9 | 52.5 | 20.3 | 34.5 | 15.2 |
| **Guideline Thyroid disorder (n=129)** | | **79.6** |  | | **5.4** | |  | **7.5** | **21.4** | **15.9** | **21.4** | **9.0** | **13.6** |  | **15.7** | **25.5** | **9.1** | **8.8** | **3.4** | **9.8** | **1.1** |
| KR9: treatment thyroid hypo function | | 94.0 |  | | 6.0 | |  | 7.6 | 12.1 | 19.6 | 4.5 | 3.8 | 12.1 |  | 18.3 | 32.9 | 5.8 | 4.6 | 2.3 | 6.1 | 0.0 |
| KR10: treatment thyroid hyper function | | 49.6 |  | | 7.0 | |  | 9.8 | 43.5 | 17.2 | 49.6 | 19.4 | 24.8 |  | 17.8 | 31.8 | 19.2 | 18.6 | 3.9 | 17.1 | 2.4 |
| KR11: referral thyroid node | | 95.3 |  | | 3.2 | |  | 5.1 | 8.6 | 11.0 | 10.1 | 3.9 | 3.9 |  | 10.9 | 11.7 | 2.4 | 3.1 | 3.9 | 6.2 | 0.8 |
| **Guideline UTI (n=120)** | | **80.0** |  | | **6.6** | |  | **14.6** | **18.7** | **27.6** | **7.5** | **8.7** | **18.4** |  | **19.5** | **25.5** | **13.4** | **8.2** | **7.1** | **11.2** | **4.8** |
| KR12: diagnosis uncomplicated UTI | | 63.3 |  | | 5.8 | |  | 16.7 | 28.4 | 34.2 | 6.7 | 17.6 | 29.4 |  | 30.0 | 25.0 | 20.3 | 14.2 | 24.1 | 16.3 | 15.0 |
| KR13: treatment uncomplicated UTI | | 90.0 |  | | 1.7 | |  | 14.6 | 5.0 | 33.4 | 1.6 | 9.2 | 6.7 |  | 20.0 | 22.5 | 7.8 | 1.7 | 0.8 | 6.1 | 1.6 |
| KR14: diagnosis complicated UTI | | 85.8 |  | | 6.6 | |  | 15.4 | 26.7 | 26.7 | 5.8 | 4.2 | 17.5 |  | 20.8 | 39.1 | 15.6 | 18.3 | 8.3 | 14.2 | 4.2 |
| KR15: treatment complicated UTI with tissue invasion | | 88.3 |  | | 4.1 | |  | 9.2 | 15.0 | 19.2 | 8.3 | 1.7 | 19.2 |  | 15.0 | 24.2 | 9.7 | 1.6 | 1.6 | 10.8 | 2.5 |
| KR16: treatment complicated UTI without tissue invasion | | 72.5 |  | | 15.0 | |  | 17.1 | 18.3 | 24.2 | 15.0 | 10.8 | 19.2 |  | 11.7 | 16.7 | 13.6 | 5.0 | 0.8 | 8.6 | 0.8 |
| **All 16 KRs** | **(mean %)** | **76.8** |  | | **9.0** | |  | **12.2** | **22.4** | **25.2** | **10.8** | **9.6** | **16.9** |  | **23.0** | **29.7** | **12.1** | **12.7** | **6.1** | **13.9** | **3.8** |
| **(SD)** | | **(15.0)** |  | | **(6.2)** | |  | **(3.75)** | **(13.4)** | **(9.1)** | **(13.0)** | **(5.3)** | **(7.5)** |  | **(15.4)** | **(9.5)** | **(6.2)** | **(14.1)** | **(7.4)** | **(9.1)** | **(5.1)** |
